# Reactivation of Hepatitis B Virus in Hematopoietic Stem Cell Transplant Recipients in Japan: Efficacy of Nucleos(t)ide Analogues for Prevention and Treatment

**DOI:** 10.3390/ijms151121455

**Published:** 2014-11-21

**Authors:** Shingo Nakamoto, Tatsuo Kanda, Chiaki Nakaseko, Emiko Sakaida, Chikako Ohwada, Masahiro Takeuchi, Yusuke Takeda, Naoya Mimura, Tohru Iseki, Shuang Wu, Makoto Arai, Fumio Imazeki, Kengo Saito, Hiroshi Shirasawa, Osamu Yokosuka

**Affiliations:** 1 Department of Molecular Virology, Graduate School of Medicine, Chiba University, 1-8-1 Inohana, Chuo-ku, Chiba 260-8677, Japan; E-Mails: nakamotoer@yahoo.co.jp (S.N.); saitok@faculty.chiba-u.jp (K.S.); sirasawa@faculty.chiba-u.jp (H.S.); 2 Department of Gastroenterology and Nephrology, Graduate School of Medicine, Chiba University, 1-8-1 Inohana, Chuo-ku, Chiba 260-8677, Japan; E-Mails: gosyou100@yahoo.co.jp (S.W.); araim-cib@umin.ac.jp (M.A.); imazekif@faculty.chiba-u.jp (F.I.); yokosukao@faculty.chiba-u.jp (O.Y.); 3 Department of Hematology, Chiba University Hospital, 1-8-1 Inohana, Chuo-ku, Chiba 260-8670, Japan; E-Mails: chiaki-nakaseko@faculty.chiba-u.jp (C.N.); esakaida@faculty.chiba-u.jp (E.S.); chikako_ohwada@faculty.chiba-u.jp (C.O.); m-takeuchi@faculty.chiba-u.jp (M.T.); take-you@hospital.chiba-u.jp (Y.T.); naoyamimura@chiba-u.jp (N.M.): iseki@faculty.chiba-u.jp (T.I.); 4 Division of Transfusion Medicine and Cell Therapy, Chiba University Hospital, 1-8-1 Inohana, Chuo-ku, Chiba 260-8670, Japan

**Keywords:** HBV, hematologic malignancy, hematopoietic stem cell, reactivation

## Abstract

We retrospectively reviewed 413 recipients with hematologic malignancies who underwent hematopoietic stem cell transplantation (HSCT) between June 1986 and March 2013. Recipients with antibody to hepatitis B core antigen (anti-HBc) and/or to hepatitis B surface antigen (anti-HBs) were regarded as experiencing previous hepatitis B virus (HBV) infection. Clinical data of these recipients were reviewed from medical records. We defined ≥1 log IU/mL increase in serum HBV DNA from nadir as HBV reactivation in hepatitis B surface antigen (HBsAg)-positive recipients, and also defined ≥1 log IU/mL increase or re-appearance of HBV DNA and/or HBsAg as HBV reactivation in HBsAg-negative recipients. In 5 HBsAg-positive recipients, 2 recipients initially not administered with nucleos(t)ide analogues (NUCs) experienced HBV reactivation, but finally all 5 were successfully controlled with NUCs. HBV reactivation was observed in 11 (2.7%) of 408 HBsAg-negative recipients; 8 of these were treated with NUCs, and fortunately none developed acute liver failure. In 5 (6.0%) of 83 anti-HBc and/or anti-HBs-positive recipients, HBV reactivation occurred. None of 157 (0%) recipients without HBsAg, anti-HBs or anti-HBc experienced HBV reactivation. In HSCT recipients, HBV reactivation is a common event in HBsAg-positive recipients, or in HBsAg-negative recipients with anti-HBc and/or anti-HBs. Further attention should be paid to HSCT recipients with previous exposure to HBV.

## 1. Introduction

Hepatitis B virus (HBV) infection is one of the major health problems in the world, causing acute and chronic hepatitis, hepatic cirrhosis and hepatocellular carcinoma (HCC) [1]. The global prevalence of hepatitis B surface antigen (HBsAg) varies [2]. In Japan, the HBV carrier rate was estimated at 0.71% in 2005 [3]. It was recommended that HBV carriers requiring immunosuppressive or cytotoxic therapy should be treated with nucleos(t)ide analogues (NUCs) [2].

Occult HBV infection is characterized by the presence of HBV DNA in the liver, and in some patients also in the serum in the absence of HBsAg [4]. In general, patients with an antibody to the hepatitis B core antigen (anti-HBc) and/or to the hepatitis B surface antigen (anti-HBs) are regarded as experiencing previous HBV infection [2], which may be related to the fact that there are no universal vaccination programs against HBV in Japan. Previous studies demonstrated that transmission of HBV could occur in recipients from anti-HBc-positive donors in living related liver transplants [5,6]. Despite having anti-HBs, chemotherapy or immunosuppression, the reactivation of HBV occurs with the reappearance of HBV DNA with or without HBsAg [7]. Thus, patients with previous exposure to HBV as well as patients currently infected with HBV face the possibility of experiencing HBV reactivation and severe liver diseases. The management of these patients, and especially those with previous exposure to HBV, is still not firmly established.

HBV infection is relatively frequent in allogeneic hematopoietic stem cell transplantation (HSCT) recipients and especially in those geographical regions where HBV infection is endemic [8]. The prevalence of HBsAg-positive patients was 0.1%–1%, 10%–30% and 22.7% in the United States, Turkey and Asia–Pacific region, respectively [8,9]. It was reported that the disappearance of anti-HBs positivity was observed in recipients from serum anti-HBs-negative donors and that these recipients faced a higher risk of HBV infection was higher in these recipients [8]. Calcineurin inhibitors such as cyclosporine and tacrolimus have improved the outcomes of HSCT [10], and rituximab, a chimeric mouse/human immunoglobulin G1 (IgG1) kappa monoclonal antibody with high affinity for CD20 antigen, which is robustly expressed by normal and malignant B cells, is also occasionally applied during the transplant treatment course [11]. However, the use of potent immunosuppressants was found to increase the probability of HBV reactivation in allogeneic HSCT recipients [12]. The balance between HBV replication and immune control after HSCT may contribute to HBV reactivation, occasionally resulting in fatal liver failure [10].

In the present study, we retrospectively examined the incidence of HBV reactivation among HSCT recipients with hematologic malignancies at our hospital in Japan, where no universal vaccination programs against HBV exist. We also evaluated the status of serum HBV markers and confirmed the effectiveness of NUCs against HBV reactivation. This study could provide new information about HBV reactivation after HSCT in HBsAg-positive and HBsAg-negative recipients.

## 2. Results

### 2.1. Hepatitis B Virus (HBV) Reactivation from Hepatitis B Surface Antigen (HBsAg)-Positive Recipients

In 120 recipients with HSCT before 2000, only 1 (1%) was HBsAg-positive and the other 119 (99%) recipients were HBsAg-negative. This single HBsAg-positive recipient experienced HBV reactivation accompanied by elevation of alanine aminotransferase (ALT) (1550 IU/L) 3 months after allogeneic HSCT for acute myeloid leukemia (AML) with cyclosporine and corticosteroid, and she was successfully treated with lamivudine and entecavir 106 months later.

In 293 recipients with HSCT in 2000 or after, 4 (1%) and 289 (99%) recipients were HBsAg-positive and -negative, respectively. Of the 4 HBsAg-positive recipients, one with autologous HSCT for multiple myeloma (MM) experienced HBV reactivation 1 month after HSCT, and he was successfully treated with lamivudine. The other 3 recipients (2 with autologous HSCT and 1 with allogeneic HSCT) were prophylactically treated with NUCs, and during the follow-up periods, up-regulation of HBV DNA levels was transiently observed, but they subsided without further treatment. In all 5 HBsAg-positive recipients, HBV DNA levels could be well controlled by the administration of NUCs.

### 2.2. HBV Reactivation from HBsAg-Negative Recipients

Of a total of 408 HBsAg-negative recipients with HSCT, 11 (2.7%) experienced HBV reactivation. In 119 HBsAg-negative recipients with HSCT before 2000 and 289 HBsAg-negative recipients with HSCT in 2000 or after, 5 (4%) and 6 (2%) recipients, respectively, experienced HBV reactivation. Among 15 (13%) HBsAg-negative recipients before 2000 and 96 (33%) HBsAg-negative recipients with autologous HSCT in 2000 or after, none had HBV reactivation. As for allogeneic HSCT recipients, 5 of 104 (5%) HBsAg-negative recipients before 2000 and 6 of 193 (3%) HBsAg-negative recipients in 2000 or after experienced HBV reactivation, respectively (no significant difference between the two periods).

### 2.3. Status of the Antibody to the Hepatitis B Surface Antigen (anti-HBs) and to the Hepatitis B Core Antigen (anti-HBc) in HBsAg-Negative Recipients with HBV Reactivation

Among 289 HBsAg-negative recipients with HSCT in 2000 or after, at least 83 (29%) had experienced previous HBV infection (Table 1). In 6 (2%) of these 289 HBsAg-negative recipients, HBV reactivation was observed: 4 (11%), 1 (2%) and 1 (2%) were observed in the groups of 35 anti-HBc-positive, 48 anti-HBc-negative/anti-HBs-positive and 49 unknown (Table 1). Cumulative HBV reactivation rates were higher in the anti-HBc-positive recipients than those in the others (*p* < 0.001, log-rank test; Table 1).

**Table 1:** Prevalence of antibodies to hepatitis B core antigen (anti-HBc) and to hepatitis B surface antigen (anti-HBs), and hepatitis B virus (HBV) reactivation rates in the 289 hepatitis B surface antigen (HBsAg)-negative recipients with hematopoietic stem cell transplantation (HSCT) in 2000 or after.

Status of Anti-HBc/Anti-HBs	Number of Patients	Number of HBV Reactivation	Cumulative HBV Reactivation Rates
(+)/(+)	27 (9%)	4 (11%)	9.1% at 2 years; 14.5% at 5 years
(+)/(−)	8 (3%)	0 (0%)	
(−)/(+)	48 (17%)	1 (2%)	0.4% at 2 years; 1.3% at 5 years
(−)/(−)	157 (54%)	0 (0%)	
NA/NA	49 (17%)	1 (2%)	

(+), positive; (−), negative; NA, not available.

### 2.4. Characteristics of HBsAg-Negative Recipients with HBV Reactivation

Characteristics of the 11 HBsAg-negative recipients with HBV reactivation after HSCT are shown in Table 2 and Table 3. Information about the donor’s HBV serology, for those with HBV reactivation, was also shown in Table 3. In 10 (91%), HBV reactivation was observed within 3 years after HSCT (4–91 months, median 19 months). In 9 (82%) of the 11, HBV reactivation occurred during immunosuppression treatment. In all 11 recipients, HBsAg reappeared. Eight of 11 recipients were treated with NUCs. Among them, 4 patients received lamivudine and 4 received entecavir. Of these 8 patients, 3 achieved HBsAg to anti-HBs seroconversion 8 to 48 months after lamivudine therapy (median 11 months), and they successfully stopped the therapy without recurrence. Another recipient achieved HBsAg clearance 15 months after entecavir therapy. These 4 recipients were treated with NUCs immediately after HBV reactivation, and achieved HBV DNA <2.6 log copies/mL with a median treatment period of 2.5 months (1–7 months) (Table 3). In the remaining 4 recipients, HBV DNA was decreased in response to NUCs therapy, but it still remained positive during the observation period. There were no serious adverse events related to NUCs therapy. Fortunately, no recipients advanced to acute liver failure, and none died due to liver diseases in the present study.

**Table 2:** Characteristics of 11 HBsAg-negative recipients with HBV reactivation after hematopoietic stem cell transplantation (HSCT).

Case	Age (Years)	Gender	Type of Disease	Transplant Type	HSCT Type	Year of HSCT	Period of Immunosuppression after HSCT (Months)	Outcome	Cause of Death	Period from HSCT to Outcome (Months)
1	42	Male	AML	Allogeneic	BMT	1988	NA	Death	Unknown	18
2	44	Male	CML	Allogeneic	BMT	1994	>20	Death	Unknown	89
3	37	Male	ALL	Allogeneic	BMT	1995	8	Death	Primary disease	188
4	46	Male	AML	Allogeneic	BMT	1997	47	Death	Renal failure	47
5	40	Female	AML	Allogeneic	PBSCT	1999	48	Death	Infection	48
6	49	Female	CML	Allogeneic	PBSCT	2000	54	Alive		163
7	49	Male	MM	Autologous/ Allogeneic	PBSCT	2000	37	Death	Primary disease	105
8	22	Male	ALL	Allogeneic	PBSCT	2004	52	Alive		109
9	54	Male	MDS	Allogeneic	BMT	2008	65	Death	Esophageal cancer	66
10	53	Female	NHL	Allogeneic	BMT	2010	31	Lost to follow-up	NA	31
11	39	Male	MF	Allogeneic	PBSCT	2005	100	Alive		100

HSCT, hematopoietic stem cell transplantation; NA, not available; AML, acute myeloid leukemia; CML, chronic myeloid leukemia; ALL, acute lymphoblastic leukemia; MM, multiple myeloma; MDS, myelodysplastic syndrome; NHL, non-Hodgkin lymphoma; MF, myelofibrosis; BMT, bone marrow transplantation; PBSCT, peripheral blood stem cell transplantation.

**Table 3:** HBV status in HBsAg-negative recipients who experienced HBV reactivation after hematopoietic stem cell transplantation (HSCT) and their donors.

Recipients (Before HSCT)			Recipients (After HSCT)								Donor	
Case	anti-HBs	anti-HBc	Last Confirmed Time of HBsAg-Negativity after HSCT (Months)	Time of HBsAg-Positivity after HSCT (Months)	HBV DNA at the Time of HBsAg-Positive (log copies/mL)	Type of NUCs	Period from HBsAg Positive to NUCs Start (Months)	Outcome of HBV Status	Period from Treatment Start to Achievement of HBV Outcome (Months)	Treatment Period (Months)	anti-HBs	anti-HBc
1	NA	NA	2	8	(+)	ND	NA	HBsAg (−)	NA	NA	NA	NA
2	NA	NA	1	18	(+)	ND	NA	HBsAg (+)	19 *	NA	NA	NA
3	NA	NA	0	33	8.2	ETV	141	HBsAg (+)	12	12	(−)	(−)
4	NA	NA	0	10	(+)	ND	NA	NA	NA	NA	(−)	(−)
5	NA	NA	0	19	7.4	LAM	24	HBsAg (+)	1	1	NA	NA
6	(+)	(+)	10	28	4.5	LAM	0	anti-HBs (+)	8	>8	NA	NA
7	(+)	NA	0	31	7.3	LAM	0	anti-HBs (+)	48	9	NA	NA
8	(+)	(−)	0	4	4	LAM	0	anti-HBs (+)	11	>11	(−)	(−)
9	(+)	(+)	5	14	8.9	ETV	41	HBsAg (+)	10	>10	(−)	(−)
10	(+)	(+)	3	20	>9	ETV	4	HBsAg (+)	6	>6	(−)	(−)
11	(+)	(+)	74	91	8.6	ETV	0	HBsAg (−)	15	>15	(−)	(−)

HSCT, hematopoietic stem cell transplantation; NUCs, nucleos(t)ide analogues; anti-HBs, antibody to hepatitis B surface antigen; anti-HBc, antibody to hepatitis B core antigen; (+), positive; (−), negative; NA, not available; ND, not done; LAM, lamivudine; ETV, entecavir.; * Period from detection of HBsAg-positive to HBV outcome.

## 3. Discussion

In the present study, the HBV reactivation rate was 100% in HBsAg-positive recipients with HSCT in the absence of HBV prophylaxis. In HBsAg-negative recipients with HSCT, 2.7% of the recipients experienced HBV reactivation. HBV reactivation was observed in 11% of HBsAg-negative and anti-HBc-positive recipients, although the number of study samples was relatively small. Notably, all recipients with HBV reactivation were successfully treated with NUCs, although some needed long-term treatment after HSCT. To add support to this conclusion, further extended follow-up periods will be needed.

In HBsAg-positive recipients, the prophylactic use of NUCs was effective for preventing HBV reactivation, supporting previous reports [13,14,15,16]. In the case of HBsAg-positive recipients, NUCs seem effective when administered with allogeneic [14] as well as with autologous HSCT [9,13]. In the present study, the 5 HBsAg-positive cases could not become NUCs-free during the follow-up periods. All patients died of primary diseases after receiving NUCs for a median period of 15 months (2–224 months). Lin *et al.* [17] reported fatal fulminant hepatitis B cases after withdrawal of prophylactic lamivudine in HSCT. Extended NUCs therapy may be safe and effective for the prevention of HBV reactivation in HBsAg-positive recipients with HSCT [18].

In HBsAg-negative recipients, we found that HBV reactivation was a rare event in both anti-HBc-negative and anti-HBs-negative recipients with HSCT. HBV reactivation occurred in almost all cases with anti-HBc and/or anti-HBs, except one case whose anti-HBc/anti-HBs status was unknown (Table 1). The status of anti-HBc and anti-HBs as well as HBsAg should be confirmed before performing HSCT, as in previous reports [19,20]. Goyama *et al.* [19] reported that the use of corticosteroids, the lack of anti-HBs in donor, and a decrease in serum anti-HBc and anti-HBs levels may predict reverse seroconversion after HSCT. Of note, HBV reactivation occurred in 82% of HBsAg-negative cases during immunosuppressive treatment in the present study. Some recent guidelines [21,22,23] have been recommended to start prophylactic antiviral therapy for HBsAg-negative recipients with anti-HBc and receiving HSCT. Tomblyn *et al.* [22] reported that, if the HSCT recipient is anti-HBc-positive and anti-HBs-positive, the risk of HBV reactivation is considered low during chemotherapy/conditioning, but it is thought to be higher following prolonged treatment with prednisone for graft-*versus*-host disease. They [22] also recommended that prophylactic antiviral treatment may be considered for anti-HBc-positive and anti-HBs-positive recipients before, and for 1 to 6 months after HSCT.

It is known that HBV vaccine could induce anti-HBs in a majority of vaccinees [24]. Because there are no universal vaccination programs against HBV in Japan, HBV infections are still viewed as an important issue [25]. Then, we should consider recipients with anti-HBs as having experienced HBV infection, perhaps a setting different from other countries where universal vaccination programs against HBV exist. HBV immunization of recipients of allogeneic HSCT results in a protective antibody response against HBV [26], and further studies concerning this issue are urgently required in our country.

Hui *et al.* [27] reported that it is uncertain whether late HBV-related hepatitis is due to *de novo* hepatitis B infection or transmission from donors. In our study, at least 6 of 11 donors for HBsAg-negative-recipients with HBV reactivation did not have anti-HBs and/or anti-HBc (Table 3). As for the frequency of follow-up of these recipients, most of them did not receive HBsAg/anti-HBs checks regularly after HSCT, or regular follow-up. As recent Japanese guidelines recommended that recipients with HBsAg, anti-HBs, or anti-HBc should receive HBsAg/anti-HBs examinations regularly after HSCT, or regular follow-up [28], we plan to perform monitoring of HBV DNA monthly for 12 months after HSCT, and monthly once per 3 months after that.

In the present study, we did not observe recipients with fatal severe liver diseases. This might be because we used NUCs in the early stage of HBV reactivation. In HBsAg-negative recipients who are anti-HBc-positive and/or anti-HBs-positive, close monitoring including the measurement of HBV DNA as well as ALT levels should be mandatory. Although the intervals of this monitoring may be discussed, the present study suggested that immediate use of NUCs might be safe and effective for the prevention of HBV reactivation in HBsAg-negative recipients with HSCT.

## 4. Patients and Methods

### 4.1. Ethics

This work was carried out in accordance with the Declaration of Helsinki (2000) of the World Medical Association. This retrospective study was approved by the Ethics Committee of Chiba University, Graduate School of Medicine on 31 January 2014 (No. 1754). Informed consent for participation in this study was obtained from all patients and/or their families by posting a notice in our institutes.

### 4.2. Patients

A total of 413 recipients (mean observation period, 25 (0–309) months), treated with HSCT at Chiba University Hospital, Chiba, Japan between June 1986 and March 2013, were retrospectively reviewed for the occurrence of HBV reactivation. The patient characteristics are shown in Table 4. The recipients were divided into two groups: recipients treated with HSCT from 1986 to 1999 (before 2000), and recipients treated with HSCT from 2000 to 2013 (in 2000 or after), as nucleic acid amplification testing (NAT) of donated blood for infectious agents was introduced in Japan in October 1999, and Japanese health insurance approved the first NUC, lamivudine, for the treatment of hepatitis B recipients in September 2000.

**Table 4:** Patient characteristics in the present study.

Characteristics	Total (*n* = 413)	1986–1999 (*n* = 120)	2000–2013 (*n* = 293)
Gender			
Male, *n* (%)	250 (60.5)	70 (58.3)	180 (61.4)
Female, *n* (%)	163 (39.5)	50 (41.7)	113 (38.6)
Median age, years (range)	42 (15–69)	32 (15–57)	47 (16–69)
Type of disease, *n* (%)			
AML	115 (27.8)	36 (30.0)	79 (26.7)
ALL	61 (14.8)	28 (23.3)	31 (10.6)
CML	38 (9.2)	20 (16.7)	18 (6.1)
MDS	31 (7.5)	9 (7.5)	22 (7.5)
Lymphoma	84 (20.3)	18 (15.0)	68 (23.2)
Plasma cell dyscrasia *^1^	61 (14.8)	0 (0)	61 (20.8)
Aplastic anemia	16 (3.9)	9 (7.5)	7 (2.4)
Others *^2^	7 (1.7)	0 (0)	7 (2.4)
Transplant type, *n* (%)			
Autologous	114 (27.6)	15 (12.5)	99 (33.8)
Allogeneic	299 (72.4)	105 (87.5)	194 (66.2)
For Allogeneic-SCT			
Donor source, *n* (%)			
Related	126 (42.1%)	59 (56.2%)	67 (34.5%)
Unrelated	135 (45.2%)	46 (43.8%)	89 (45.9%)
Unrelated cord blood	38 (12.7%)	0 (0%)	38 (19.6%)

AML, acute myeloid leukemia; ALL, acute lymphoblastic leukemia; CML, chronic myeloid leukemia; MDS, myelodysplastic syndrome; SCT, stem cell transplantation. *^1^ Plasma cell dyscrasia included multiple myeloma, AL amyloidosis and POEMS syndrome; *^2^ Others included primary myelofibrosis, chronic active EB virus infection and chronic eosinophilic leukemia.

### 4.3. Serological Examination

HBsAg, anti-HBs and anti-HBc were determined by ELISA or chemiluminescent enzyme immunoassay (CLEIA) [29]. Depending on the time-point during this retrospective study, HBV DNA was measured by Roche Amplicore PCR assay (detection limit: 2.6 log IU/mL), COBAS TaqMan HBV test v2.0 (detection limit: 2.0 log IU/mL) (Roche Diagnostics, Basel, Switzerland), or PCR methods. If needed, we performed in-house nested PCR [29]. All serological tests were performed at the Central Laboratory of Chiba University Hospital.

**Figure 1:**
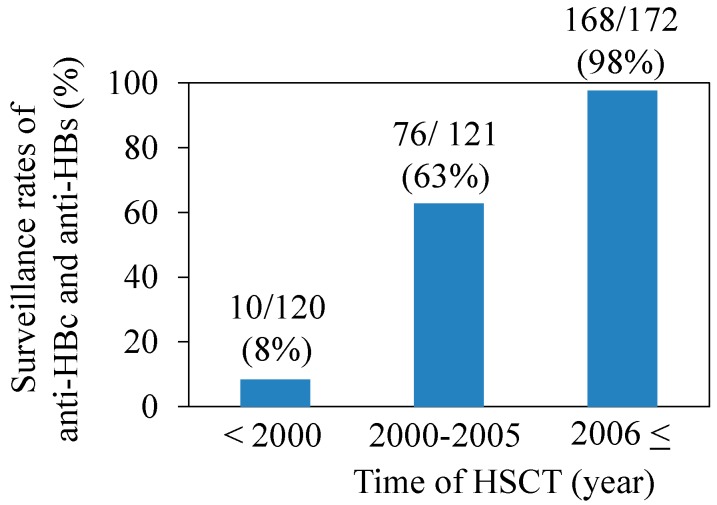
Surveillance rates of antibodies to hepatitis B core antigen (anti-HBc) and to hepatitis B surface antigen (anti-HBs) according to the time of hematopoietic stem cell transplantation (HSCT).

HBsAg was measured in all recipients before and after HSCT. Among 120 recipients with HSCT before 2000, anti-HBs and anti-HBc were measured in only 10 (8%) recipients. Among the patients with HSCT performed in 2000 or after, the recipients were further divided into two groups: 2000–2005 and 2006–2013. Of 121 recipients with HSCT in 2000–2005, anti-HBs and anti-HBc were measured in 76 (63%) recipients. Finally, among 172 recipients with HSCT in 2006–2013, anti-HBs and anti-HBc were measured in 168 (98%) recipients (Figure 1). HBsAg, anti-HBs and anti-HBc were measured for screening in almost all recipients after the Japanese Health and Labor Sciences Research Group for “Clarification of current status for reactivation of hepatitis B virus associated with immunosuppressants and antineoplastics and establishment of preventive measures” started a registry in 2009 for HBV-infected patients with hematopoietic malignancies [28].

### 4.4. Definition of HBV Infection Status and HBV Reactivation

In the present study, before HSCT, the recipients were divided into two groups according to HBsAg: HBsAg-positive and HBsAg-negative. Among the HBsAg-negative recipients, anti-HBc-positive and/or anti-HBs-positive recipients were considered as having experienced previous HBV infection [2], and recipients without anti-HBc and anti-HBs were considered as having no previous or current HBV infection.

We defined ≥1 log IU/mL increase in serum HBV DNA from nadir as HBV reactivation in HBsAg-positive recipients. We also defined ≥1 log IU/mL increase, or the re-appearance of HBV DNA from baseline and/or HBsAg, as HBV reactivation in HBsAg-negative recipients.

### 4.5. Statistical Analysis

Statistical analyses were performed using Statview-J 5.0 (SAS institute, Cary, NC, USA). HBV reactivation rates were calculated by Kaplan-Meier method and evaluated by log-rank test. Baseline was taken as the date of HSCT. *p* < 0.05 was considered statistically significant.

## 5. Conclusions

HBV reactivation was a common event in HBsAg-positive recipients with HSCT for hematologic malignancies, NUCs are safely and effectively used in these recipients, and extended NUCs therapy may be needed for the prevention of HBV reactivation. In addition, HBV reactivation was occasionally observed in HBsAg-negative recipients with anti-HBc and/or anti-HBs and treated with HSCT, and the immediate use of NUCs could prevent the progression to severe liver damage. Special attention should be paid to recipients with previous exposure to HBV.

## References

[1] Di Bisceglie A.M. (2009). Hepatitis B and hepatocellular carcinoma. Hepatology.

[2] Lok A.S., McMahon B.J. (2007). Chronic hepatitis B. Hepatology.

[3] Tanaka J., Koyama T., Mizui M., Uchida S., Katayama K., Matsuo J., Akita T., Nakashima A., Miyakawa Y., Yoshizawa H. (2011). Total numbers of undiagnosed carriers of hepatitis C and B viruses in Japan estimated by age- and area-specific prevalence on the national scale. Intervirology.

[4] Ramimondo G., Pollicino T., Romano L., Zanetti A.R. (2010). A 2010 update on occult hepatitis B infection. Pathol. Biol..

[5] Uemoto S., Sugiyama K., Marusawa H., Inomata Y., Asonuma K., Egawa H., Kiuchi T., Miyake Y., Tanaka K., Chiba T. (1998). Transmission of hepatitis B virus from hepatitis B core antibody-positive donors in living donors in living related liver transplants. Transplantation.

[6] Marusawa H., Uemoto S., Hijikata M., Ueda Y., Tanaka K., Shimotohno K., Chiba T. (2000). Latent hepatitis B infection in healthy individuals with antibodies to hepatitis B core antigen. Hepatology.

[7] Palmore T.N., Shah N.L., Loomba R., Borg B.B., Lopatin U., Feld J.J., Khokhar F., Lutchman G., Kleiner D.E., Young N.S. (2009). Reactivation of hepatitis B with reappearance of hepatitis B surface antigen after chemotherapy and immunosuppression. Clin. Gastroenterol. Hepatol..

[8] Ustün C., Koç H., Karayalcin S., Akyol G., Gürman G., Ilhan O., Akan H., Ozcan M., Arslan O., Konuk N. (1997). Hepatitis B virus infection in allogeneic bone marrow transplantation. Bone Marrow Transplant..

[9] Ma S.Y., Lau G.K., Cheng V.C., Liang R. (2003). Hepatitis B reactivation in patients positive for hepatitis B surface antigen undergoing autologous hematopoietic cell transplantation. Leuk. Lymphoma.

[10] Caselitz M., Link H., Hein R., Maschek H., Böker K., Poliwoda H., Manns M.P. (1997). Hepatitis B associated liver failure following bone marrow transplantation. J. Hepatol..

[11] Naparstek E. (2006). The role of rituximab in autologous and allogeneic hematopoietic stem cell transplantation for non-Hodgkin’s lymphoma. Curr. Hematol. Malig. Rep..

[12] Mikulska M., Nicolini L., Signori A., Rivoli G., del Bono V., Raiola A.M., di Grazia C., Dominietto A., Varaldo R., Ghiso A. (2014). Hepatitis B reactivation in HBsAg-negative/HBcAb-positive allogeneic haematopoietic stem cell transplant recipients: Risk factors and outcome. Clin. Microbiol. Infect..

[13] Endo T., Sakai T., Fujimoto K., Yamamoto S., Takashima H., Haseyama Y., Nishio M., Koizumi K., Koike T., Sawada K. (2001). A possible role for lamivudine as prophylaxis against hepatitis B reactivation in carriers of hepatitis B who undergo chemotherapy and autologous peripheral blood stem cell transplantation for non-Hodgkin's lymphoma. Bone Marrow Transplant..

[14] Henkes M., Martin S., Einsele H., Aulitzky W.E. (2002). Successful antiviral treatment for fulminant reactivated hepatitis B after autologous stem cell transplantation and prophylaxis during subsequent allogeneic stem cell transplantation. Ann. Hematol..

[15] Rossi G. (2003). Prophylaxis with lamivudine of hepatitis B virus reactivation in chronic HbsAg carriers with hemato-oncological neoplasias treated with chemotherapy. Leuk. Lymphoma.

[16] Milazzo L., Corbellino M., Foschi A., Micheli V., Dodero A., Mazzocchi A., Montefusco V., Zehender G., Antinori S. (2012). Late onset of hepatitis B virus reactivation following hematopoietic stem cell transplantation: successful treatment with combined entecavir plus tenofovir therapy. Transpl. Infect. Dis..

[17] Lin P.C., Poh S.B., Lee M.Y., Hsiao L.T., Chen P.M., Chiou T.J. (2005). Fatal fulminant hepatitis B after withdrawal of prophylactic lamivudine in hematopoietic stem cell transplantation patients. Int. J. Hematol..

[18] Hsiao L.T., Chiou T.J., Liu J.H., Chu C.J., Lin Y.C., Chao T.C., Wang W.S., Yen C.C., Yang M.H., Tzeng C.H. (2006). Extended lamivudine therapy against hepatitis B virus infection in hematopoietic stem cell transplant recipients. Biol. Blood Marrow Transplant..

[19] Goyama S., Kanda Y., Nannya Y., Kawazu M., Takeshita M., Niino M., Komeno Y., Nakamoto T., Kurokawa M., Tsujino S. (2002). Reverse seroconversion of hepatitis B virus after hematopoietic stem cell transplantation. Leuk. Lymphoma.

[20] Onozawa M., Hashino S., Izumiyama K., Kahata K., Chuma M., Mori A., Kondo T., Toyoshima N., Ota S., Kobayashi S. (2005). Progressive disappearance of anti-hepatitis B surface antigen antibody and reverse seroconversion after allogeneic hematopoietic stem cell transplantation in patients with previous hepatitis B virus infection. Transplantation.

[21] Zaia J., Baden L., Boeckh M.J., Chakrabarti S., Einsele H., Ljungman P., McDonald G.B., Hirsch H., Center for International Blood and Marrow Transplant Research, National Marrow Donor Program (2009). Viral disease prevention after hematopoietic cell transplantation. Bone Marrow Transplant..

[22] Tomblyn M., Chiller T., Einsele H., Gress R., Sepkowitz K., Storek J., Wingard J.R., Young J.A., Boeckh M.A. (2009). Guidelines for preventing infectious complications among hematopoietic cell transplant recipients: A global perspective. Bone Marrow Transplant..

[23] Reddy N.M., Savani B.N. (2013). Hepatitis B reactivation in patients with hematological malignancies and stem cell transplantation. J. Blood Lymph.

[24] Beasley R.P., Hwang L.Y., Lee G.C., Lan C.C., Roan C.H., Huang F.Y., Chen C.L. (1983). Prevention of perinatally transmitted hepatitis B virus infections with hepatitis B immune globulin and hepatitis B vaccine. Lancet.

[25] Yan J., Kanda T., Wu S., Imazeki F., Yokosuka O. (2012). Hepatitis A, B, C and E virus markers in Chinese residing in Tokyo, Japan. Hepatol. Res..

[26] Idilman R., Ustün C., Karayalçin S., Aktemel A., Turkyilmaz A.R., Ozcan M., Arslan O., Bozdayi A.M., van Thiel D.H., Akan H. (2003). Hepatitis B virus vaccination of recipients and donors of allogeneic peripheral blood stem cell transplantation. Clin. Transplant..

[27] Hui C.K., Lie A., Au W.Y., Leung Y.H., Ma S.Y., Cheung W.W., Zhang H.Y., Chim C.S., Kwong Y.L., Liang R. (2005). A long-term follow-up study on hepatitis B surface antigen-positive patients undergoing allogeneic hematopoietic stem cell transplantation. Blood.

[28] Oketani M., Ido A., Uto H., Tsubouchi H. (2012). Prevention of hepatitis B virus reactivation in patients receiving immunosuppressive therapy or chemotherapy. Hepatol. Res..

[29] Kamezaki H., Kanda T., Wu S., Nakamoto S., Arai M., Maruyama H., Fujiwara K., Imazeki F., Yokosuka O. (2011). Emergence of entecavir-resistant mutations in nucleos(t)ide-naive Japanese patients infected with hepatitis B virus: Virological breakthrough is also dependent on adherence to medication. Scand. J. Gastroenterol..

